# Ginsenoside Rb3 Inhibits Pro-Inflammatory Cytokines via MAPK/AKT/NF-κB Pathways and Attenuates Rat Alveolar Bone Resorption in Response to *Porphyromonas gingivalis* LPS

**DOI:** 10.3390/molecules25204815

**Published:** 2020-10-20

**Authors:** Minmin Sun, Yaoting Ji, Zhen Li, Rourong Chen, Shuhui Zhou, Chang Liu, Minquan Du

**Affiliations:** The State Key Laboratory Breeding Base of Basic Science of Stomatology (Hubei MOST) & Key Laboratory of Oral Biomedicine Engineering Ministry of Education, School & Hospital of Stomatology, Wuhan University, Wuhan 430079, China; sunminmin@whu.edu.cn (M.S.); yaotingji@whu.edu.cn (Y.J.); lizhen666@whu.edu.cn (Z.L.); chenrourong@whu.edu.cn (R.C.); zshc07@163.com (S.Z.)

**Keywords:** anti-inflammatory agents, chronic periodontitis, ginsenoside, lipopolysaccharides

## Abstract

Conventional treatments for chronic periodontitis are less effective in controlling inflammation and often relapse. Therefore, it is necessary to explore an immunomodulatory medication as an adjuvant. Ginsenoside Rb3 (Rb3), one of the most abundant active components of ginseng, has been found to possess anti-inflammatory and immunomodulatory properties. Here, we detected the anti-inflammatory effect of Rb3 on *Porphyromonas gingivalis* LPS-stimulated human periodontal ligament cells and experimental periodontitis rats for the first time. We found that the expression of pro-inflammatory mediators, including IL-1β, IL-6 and IL-8, upregulated by lipopolysaccharide (LPS) stimulation was remarkably downregulated by Rb3 treatment in a dose-dependent manner at both transcriptional and translational levels. Network pharmacological analysis of Rb3 showed that the mitogen-activated protein kinase (MAPK) signaling pathway had the highest richness and that p38, JNK, and ERK molecules were potential targets of Rb3 in humans. Western blot analysis revealed that Rb3 significantly suppressed the phosphorylation of p38 MAPK and p65 NF-κB, as well as decreased the expression of total AKT. In experimental periodontitis rat models, reductions in alveolar bone resorption and osteoclast generation were observed in the Rb3 treatment group. Thus, we can conclude that Rb3 ameliorated *Porphyromonas gingivalis* LPS-induced inflammation by inhibiting the MAPK/AKT/NF-κB signaling pathways and attenuated alveolar bone resorption in experimental periodontitis rats.

## 1. Introduction

Periodontitis is characterized by an aberrant inflammatory response to oral commensal biofilms, resulting in destruction of periodontal tissues, especially in susceptible populations [1]. Periodontitis is also associated with a series of systemic diseases, such as endocarditis [2], cerebral vascular disease [3], and diabetes [4]. 

*Porphyromonas gingivalis* (*P. gingivalis*) is a major periodontal pathogenic bacterium. Its lipopolysaccharide (LPS) plays a crucial role in the pathogenesis of periodontitis by stimulating the host to secrete various pro-inflammatory mediators [5,6,7]. Human periodontal ligament cells (HPLCs), the main cellular constituents of the periodontium, secrete pro-inflammatory cytokines, such as interleukin (IL)-1β, IL-6, and IL-8, in response to pathogens, such as *P. gingivalis* [8]. The roles of nuclear factor-κB (NF-κB) and mitogen-activated protein kinase (MAPK) signaling pathways are well established in the inflammatory response of periodontal tissues as well as the damage and formation of alveolar bone [9,10]. The protein kinase B (AKT) pathway is also reported to be involved in inflammation regulation [11]. 

To date, the treatment of chronic periodontitis mainly includes subgingival curettage and other physical methods to remove plaque biofilms. However, traditional treatments are less effective in controlling inflammatory damage, and even the inflammation often relapses. Therefore, it is necessary to explore an immunomodulatory medication as an adjuvant to conventional mechanical treatment. *Panax notoginseng* has been used in China for thousands of years [12]. A systematic review evaluating the therapeutic effects of ginseng in clinical randomized controlled trials concluded that ginseng had potential therapeutic effects in terms of immune response [13]. Ginsenoside Rb3 (Rb3) is one of the most abundant and active components of ginseng. Rb3 has been reported to exert protective effects against cardiomyocyte ischemia-reperfusion injury [14], cisplatin-induced nephrotoxicity [15], and endothelial dysfunction [16] by inhibiting inflammatory responses. However, no studies have been performed concerning the anti-inflammatory effect of Rb3 on periodontitis until now.

The present study detected the anti-inflammatory effect of Rb3 on both HPLCs and experimental periodontitis rats in response to *P. gingivalis* LPS, and further explored the underlying molecular mechanisms. This research will help to better understand the pharmacological mechanism of Rb3, and offer theoretical evidence for the adjuvant immunomodulation therapy to conventional mechanical therapy in periodontitis treatment.

## 2. Results

### 2.1. Characterization of Primary HPLCs

**Figure 1:**
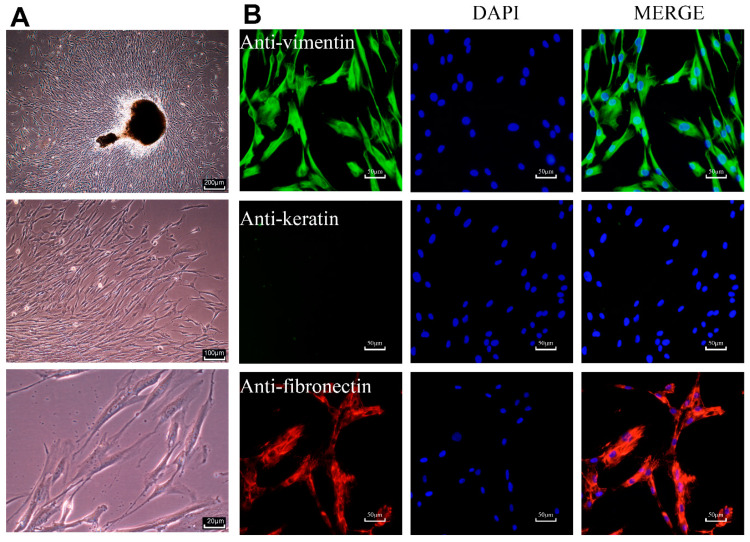
Cultivation and characterization of primary human periodontal ligament cells (HPLCs). (**A**) Cultured primary HPLCs observed under optical microscope. (**B**) HPLCs were characterized by anti-vimentin, anti-fibronectin and anti-keratin antibodies. The tested cells were positive for vimentin (green fluorescent) and fibronectin (red fluorescent) and negative for keratin (green fluorescent which was absent).

Primary HPLCs were typically spindle-shaped under light microscopy (Figure 1A). Immunofluorescence staining images revealed the positive expression of the mesenchymal markers, vimentin and fibronectin, as well as the absence of epithelial marker, keratin, which further confirmed that they originated from mesenchymal stem cells (Figure 1B). 

### 2.2. Rb3 Attenuates the Expression of IL-6, IL-8 and IL-1β in Response to P. gingivalis LPS

**Figure 2:**
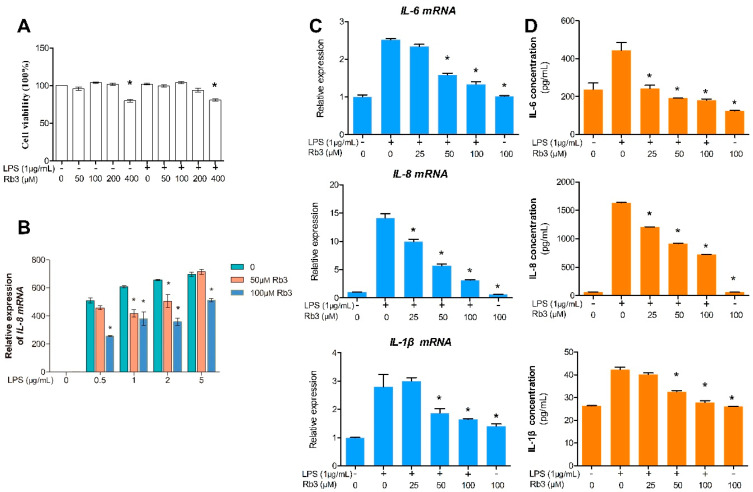
Rb3 attenuates expression of IL-6, IL-8 and IL-1β in response to *P. gingivalis* lipopolysaccharide (LPS). (**A**) Effect of Rb3 on HPLCs viability was tested using CCK-8 assay. Data were presented as means ± SE (n = 6). * *p* < 0.05 compared with normal control group. (**B**) Relative levels of *IL-8 mRNA* (24 h) determined by qPCR. (**C**) Relative levels of *IL-6, IL-8* (24 h) and *IL-1β* (4 days) *mRNA* determined by qPCR. (**D**) Proteins levels of IL-6, IL-8 (24 h) and IL-1β (4 days) in supernatants were measured using enzyme-linked immunosorbent (ELISA). (**B**–**D**) Data were presented as means ± SE (n = 3), * *p* < 0.05 compared with LPS stimulation only group. SE, standard error.

Cell counting kit-8 (CCK-8) assay was performed to determine the cytotoxicity of Rb3 at different doses. HPLCs were treated with 50, 100, 200, and 400 μM Rb3 with or without LPS stimulation for 24 h. The CCK-8 assay demonstrated that Rb3 had no effect on HPLCs viability at concentrations of 50, 100, and 200 μM, while HPLCs viability was decreased at 400 μM Rb3 with or without LPS stimulation compared with the normal control group (Figure 2A). The data in Figure 2B showed that *P. gingivalis* LPS (0.5, 1, 2 or 5 μg/mL) significantly induced IL-8 mRNA production in HPLCs in a dose-dependent manner, and this induction was attenuated by Rb3 pretreatment to different extents. *P. gingivalis* LPS at a concentration of 1 μg/mL was chosen as an appropriate stimulant in this study according to the above data. Enzyme-linked immunosorbent (ELISA) and quantitative polymerase chain reaction (qPCR) assays showed that the expression of IL-6, IL-8 and IL-1β was remarkably upregulated by *P. gingivalis* LPS, and that Rb3 pretreatment significantly downregulated the enhancement at both transcriptional and translational levels in a dose-dependent manner. (Figure 2C,D).

### 2.3. Potential Target Candidates Analysis of Rb3

**Figure 3:**
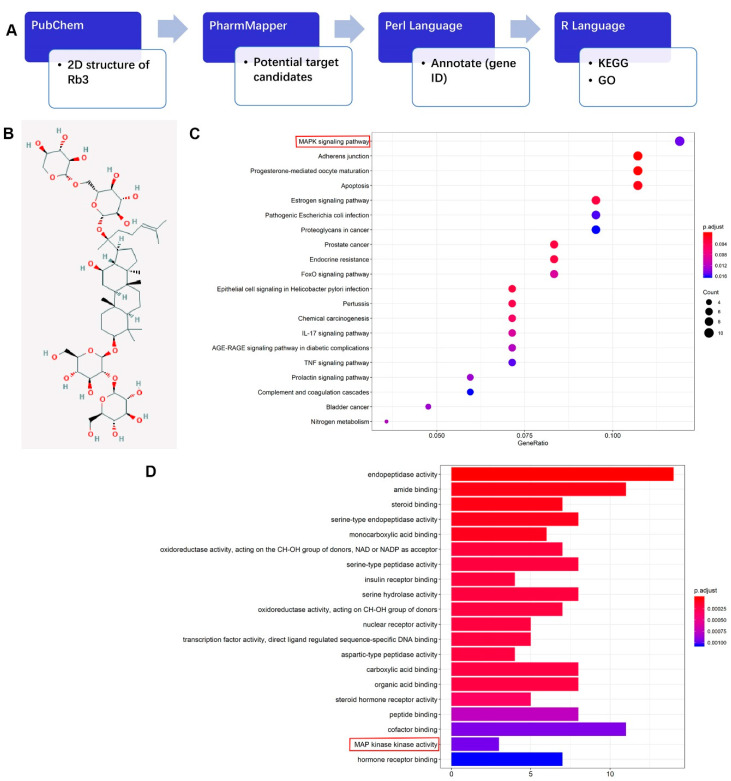
Network pharmacological analysis for Rb3. (**A**) The overall workflow of network pharmacological analysis. (**B**) The chemical structural formula of Rb3. (**C**) Top 20 enrichment of pathways related to Rb3 from GO enrichment analysis. (**D**) Top 20 active proteins interact with Rb3 from KEGG analysis.

To further explore the underlying molecular mechanisms, a network pharmacological method was conducted to predict the potential targets of Rb3 in humans. The analysis was performed based on the chemical structures and the pharmacophore models of Rb3 by means of several programming languages and databases, which are shown in the flowchart in Figure 3A. We obtained the two-dimensional structure of Rb3 from the Pubchem website, and uploaded the chemical structure on the PharmMapper website, which provided the pharmacophore models and potential protein target candidates of Rb3 according to the bond forces between molecules. Perl Language was then applied to annotate these protein candidates with gene ID. Finally, GO and KEGG analyses were performed based on the abovementioned information with R Language. GO analysis concluded that the MAPK signaling pathway was the most closely linked pathway to Rb3 (Figure 3C), and that p38, c-Jun N-terminal kinase (JNK) and extracellular signal-regulated kinase (ERK) molecules in the MAPK signaling pathways were potential targets of Rb3 in humans (Figure S1). KEGG analysis showed that MAP kinase activity was highly enriched on Rb3 (Figure 3D).

### 2.4. Rb3 Suppresses Inflammatory Activation of p38 MAPK, AKT and NF-kB

**Figure 4:**
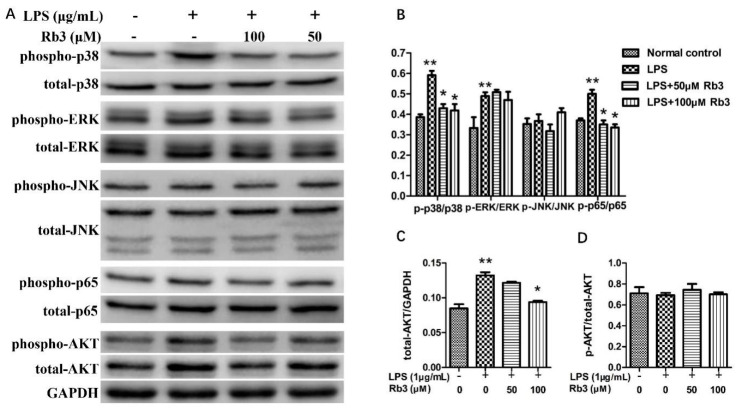
Rb3 suppresses inflammatory activation of p38 MAPK, AKT, and NF-κB. HPLCs were pretreated with Rb3 for 60 min and stimulated with *P. gingivalis* LPS for 30 min. (**A**–**D**) Detection and quantification of proteins expression by Western blot and Image J analysis. Data were presented as means ± SE (n = 3). SE, standard error. * *p* < 0.05 compared with LPS stimulation only group. ** *p* < 0.05 compared with normal control.

By combining network pharmacology analysis results and previous studies of other researchers on inflammatory diseases [9,10,11], we examined the effect of Rb3 on the MAPKs, AKT, and NF-kB pathways in *P. gingivalis* LPS-stimulated HPLCs. Western blot analyses showed that *P. gingivalis* LPS stimulation significantly increased the phosphorylation of p38 MAPK, ERK, and p65 NF-κB molecules but that it had no effect on JNK (Figure 4A,B). Rb3 treatment decreased the phosphorylation of p38 MAPK and p65 NF-κB but had little effect on ERK and JNK in *P. gingivalis* LPS-stimulated HPLCs (Figure 4A,B). Moreover, Rb3 significantly suppressed the expression of total-AKT and phospho (p)-AKT, which were upregulated by LPS stimulation (Figure 4A,C); however, the ratio of p-AKT/total-AKT was unchanged (Figure 4D), which indicated that Rb3 affected AKT signaling by downregulating the production of total-AKT rather than enhancing the phosphorylation of AKT.

### 2.5. Rb3 Inhibits Chronic Inflammation by Downregulating the Expression of Toll-like Receptor (TLR)2

**Figure 5:**
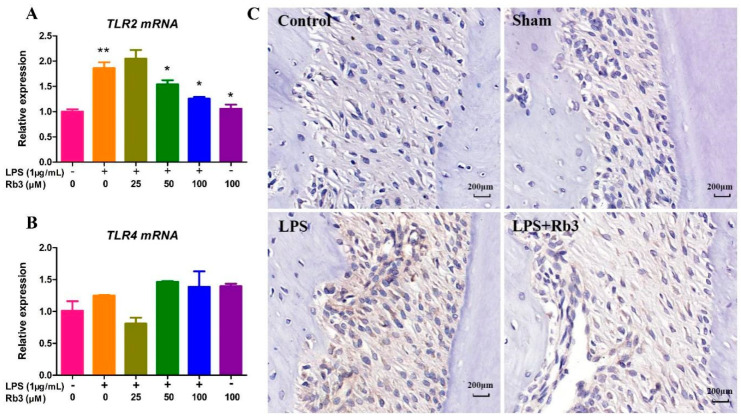
Rb3 inhibits chronic inflammation by downregulating expression of TLR2. (**A**,**B**) The relative levels of TLR2 and TLR4 mRNA (4 days) in HPLCs were examined by qPCR. (**C**) Expression of TLR2 in periodontal tissues of rat was detected by IHC analysis. Scale bar represents 20 μm. All data were presented as means ± SE (n = 3). SE, standard error. * *p* < 0.05 significantly different from LPS stimulation only group. ** *p* < 0.05 compared with normal control.

QPCR analysis showed that *P. gingivalis* LPS significantly enhanced TLR2 mRNA expression after four days of stimulation but had little effect on TLR4 mRNA expression (Figure 5A,B). The expression of TLR2 mRNA was significantly downregulated by Rb3 pretreatment (50 and 100 μM) in a dose-dependent manner in response to *P. gingivalis* LPS (Figure 5A), which was confirmed by immunohistochemical (IHC) analysis, showing that the expression of TLR2 was much higher in the LPS group than the control, sham, and LPS + Rb3 groups (Figure 5C). 

### 2.6. Rb3 Treatment Ameliorates Alveolar Bone Resorption in Rats

**Figure 6:**
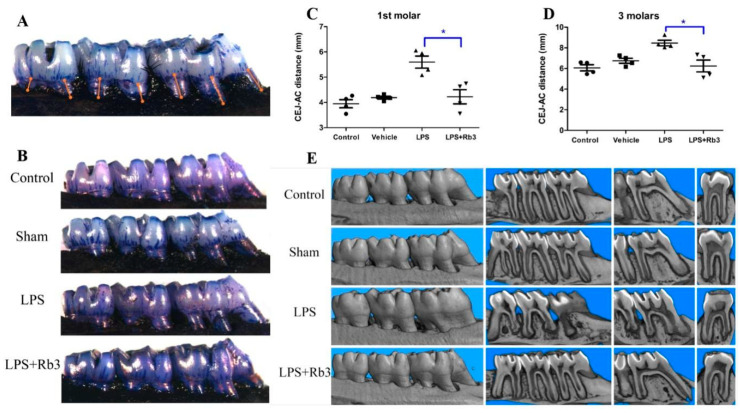
Rb3 treatment ameliorates alveolar bone resorption in rats. (**A**) Digital image indicating 7 measurement sites of the right half maxilla from the palatal aspect. (**B**) Stereomicroscopic images of the right jaw from palatal direction, CEJ and AC were delineated by methylene blue. (**C**) Statistical analysis of ABL for the first molar was presented in scatter graph. (**D**) Statistical analysis of ABL for all three molars was presented in scatter graph. (**E**) Micro-CT 3D reconstruction images and cross-section images of rats. Results were presented as means ± SE (n = 4 rats/group). * *p* < 0.05 versus LPS group. ABL, alveolar bone loss; CEJ, cemento-enamel junction; AC, alveolar bone; SE, standard error.

Seven palatal distances from the cemento-enamel junction (CEJ) and alveolar crest (AC) were measured to analyze alveolar bone loss (ABL, Figure 6A). *P. gingivalis* LPS-injected rats presented more bone loss than rats of the other three groups (control, sham, and LPS + Rb3), and no significant differences were observed between the control group and sham group (Figure 6B). Rb3 treatment significantly decreased ABL induced by LPS stimulation in the first molar and all three molar teeth (Figure 6C,D). This effect was also observed in micro-CT analysis (Figure 6E). The 3-dimensional reconstruction of rat jaws (first column of Figure 6E) from the four experimental groups showed that the alveolar ridges in the control group, sham group, as well as LPS + Rb3 group were in the form of waves but that the regular waves disappeared and presented a depressed shape instead in the LPS group. The coronal plane of rat molars (second column of Figure 6E) demonstrated that the alveolar bone between the first and second as well as between the second and third molars in the LPS group exhibited obvious resorption but that little resorption occurred in the control, sham, and LPS + Rb3 groups. The third and fourth columns of Figure 6E display a wider periodontal space and more severe interradicular alveolar bone resorption in the first molar in the LPS group than in the other three groups (control, sham, and LPS + Rb3). Figure 6E suggests that LPS injection induced compact bone resorption and that this effect was inhibited by Rb3 treatment.

### 2.7. Rb3 Attenuates P. gingivalis LPS-Induced Osteoclast Differentiation

**Figure 7:**
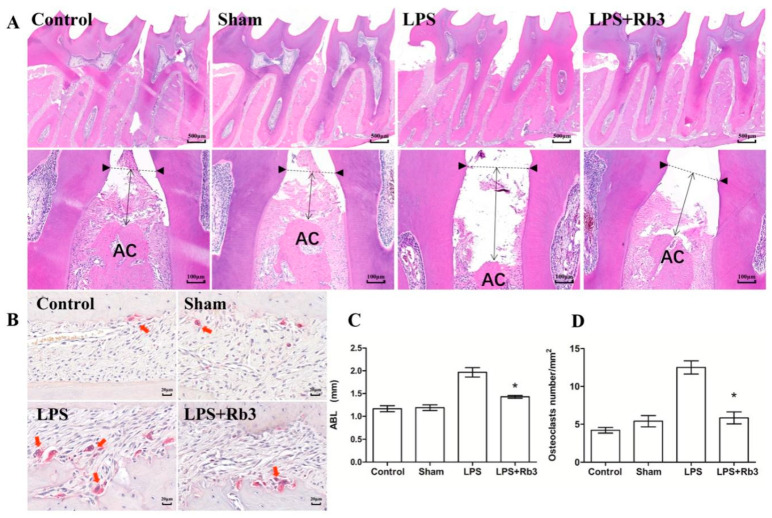
Rb3 attenuated *P. gingivalis* LPS-induced osteoclast differentiation and bone resorption in rats. (**A**) ABL was compared from H&E staining slides. Black triangles represent CEJ, and distance from CEJ to AC was measured between first-second molars of rats. (**B**) Tartrate-resistant acid phosphatase (TRAP) staining for maxilla slides of rats. Red staining cells (arrows) are osteoclasts and their immediate precursors. (**C**) ABL were calculated and presented in bar graph. (**D**) Osteoclasts were enumerated and presented in bar graph. Data were presented as means ± SE (n = 4 rats/group). * *p* < 0.05 versus LPS group. CEJ, cemento-enamel junction; ABL, alveolar bone loss; AC, alveolar bone; SE, standard error.

Histomorphometric analysis of hematoxylin and eosin (H&E)-stained sections indicated that *P. gingivalis* LPS-injected SD rats without Rb3 treatment presented more bone loss than rats in the control, sham, and LPS + Rb3 groups (Figure 7A). The bar graph in Figure 7C shows that LPS stimulation significantly enhanced alveolar bone resorption and that this effect was suppressed by Rb3 treatment. Tartrate-resistant acid phosphatase (TRAP) staining images demonstrated more osteoclast genesis in the LPS group than in the other groups (Figure 7B). The alveolar bone of *P. gingivalis* LPS-injected rats showed a statistically higher osteoclast number per square millimeter, and this ratio was significantly lower in the LPS + Rb3 group than in the LPS group (Figure 7D).

## 3. Discussion

Numerous studies have pointed out that ginseng exerts some pharmacological effects via anti-inflammatory properties [12,17]. Among the most abundant and active components of ginseng, Rb3 is water soluble and less cytotoxic, and Rb3 has been reported to exert anti-inflammatory [14], antioxidant [18], anti-depressant [19], anti-autophagy, and anti-apoptosis [15] effects via certain signaling pathways, such as JNK/NF-κB [14] or PERK/Nrf2/HMOX1 [18]. A previous study has demonstrated that Rb3 inhibits the expression of pro-inflammatory factors, such as IL-8 and tumor necrosis factor alpha in fibroblasts and epithelial cells by inhibiting the phosphorylation of p38 MAPK and NF-κB [20]. An animal study has also shown that Rb3 inhibits the secretion of pro-inflammatory cytokines after cerebral ischemia reperfusion injury in rats [21]. Consistent with the above studies, we found that Rb3 significantly suppressed the production of pro-inflammatory mediators, including IL-6, IL-8, and IL-1β. Activation of the immune response in periodontal tissues may initially be intended to protect; however, inflammatory cytokines may become destructive to tissues when they fail to limit and resolve early infection in a timely manner in susceptible individuals [22,23]. In this manner, suppression of the immune response may be the direction of immunotherapy for chronic periodontitis.

HPLCs, as the main functional cells in the periodontium, are involved in the immune response by secreting a series of chemokines and cytokines [24,25]. In the present study, we found that HPLCs produced large amounts of IL-8 in a short time after LPS stimulation, which was in accordance with a previous study [26]. Since IL-8 is a strong neutrophil chemoattractant and activator [27], HPLCs may work as a frontline of periodontal tissues by recruiting immune cells as soon as confronted with foreign invasion.

IL-1β is a pro-inflammatory cytokine involved in the pathogenesis of periodontal disease related to inflammation, connective tissue breakdown, and bone loss [28]. We found that there was little IL-1β expressed in HPLCs stimulated by *P. gingivalis* LPS until the stimulation time was prolonged to four days, which was inconsistent with several previous studies [29,30]. Mature IL-1β is a product of the inflammasome signaling pathway, which requires a second messenger, such as ATP [31]; therefore, IL-1β may play a crucial role in the chronic process of periodontitis and cannot be used as an inflammation marker when the stimulation time is short.

Numerous studies affirmed the important roles of MAPKs and NF-κB signaling pathways in periodontitis [9,10]. Our experiment showed that *P. gingivalis* LPS significantly enhanced the phosphorylation of p38 MAPK and p65 NF-κB. Moreover, we found that the expression of total-AKT was increased by *P. gingivalis* LPS stimulation, which indicated that the AKT signaling pathway may also play a role in the periodontal inflammation process. Rb3 pretreatment decreased the phosphorylation of p38 MAPK and p65 NF-κB, as well as the expression of total-AKT induced by *P. gingivalis* LPS, suggesting that Rb3 may exert anti-inflammatory effects by inhibiting the MAPK/AKT/NF-κB signaling pathways.

TLRs have been suggested to be a driving factor in periodontal disease by exaggerating inflammation [32]. A debate about what is the binding receptor for *P. gingivalis* LPS among TLRs has been conducted for decades. Previous studies have demonstrated that *P. gingivalis* or *P. gingivalis* components activate cells mainly through TLR2 [33,34,35,36,37]. However, some studies have suggested that *P. gingivalis* LPS also has activity for TLR4 [38,39,40]. We found *P. gingivalis* LPS significantly enhanced TLR2 mRNA but had little effect on TLR4 mRNA expression, which was consistent with an in vitro study demonstrating that *P. gingivalis* (whole bacteria) induces the upregulation of TLR2 with little change in the TLR4 mRNA expression level [41]. We also found that the enhancement of TLR2 mRNA induced by 4 days of *P. gingivalis* LPS stimulation was downregulated by Rb3 treatment. Thus, the expression of TLR2 was enhanced to indirectly promote the inflammatory process, with the secretion of various pro-inflammatory factors response to *P. gingivalis* LPS, and Rb3 ameliorated this chronic inflammatory process by downregulating TLR2 expression. 

In the present study, we chose to set up an experimental periodontitis rat model by gingival injection rather than ligature although the latter is more classical and popular. Because foreign bodies in the ligature model prevent the attenuation of the inflammatory process after treatments, the LPS-induced periodontitis model is more appropriate for the study of potential therapies [42]. 

We confirmed the anti-inflammatory effect of Rb3 in HPLCs and in SD rats; however, more details about the involved signaling pathways should be further verified by blocking the potential molecules.

## 4. Materials and Methods

### 4.1. Primary Culture of HPLCs

This study was carried out following the rules of the Declaration of Helsinki of 1975 revised in 2013 and approved by the Ethics Committee of School of Stomatology, Wuhan University, China (201767). HPLCs were obtained from healthy third molars extracted from six patients (four males and two females, aged 18 to 21 years) who required tooth extraction. Periodontal ligaments were cut and incubated in DMEM (Hyclone, Logan, UT, USA) at 37 °C with 5% CO_2_. When they reached 80~90% confluence, cells were sub-cultured at a 1:2 split ratio. After 3~4 subcultures with trypsinization, homogeneous, slim, and spindle-shaped cells growing in characteristic swirls were observed under ordinary optical microscope (Figure 1A), and cells from passages 4~7 were used for subsequent experiments. 

### 4.2. Cell Immunofluorescence Assay

Primary HPLCs were characterized by examining the expression of mesenchymal marker vimentin, fibronectin, and epithelial marker keratin. Cells at the fourth passage were fixed, permeabilized, and incubated with primary antibodies recognizing vimentin, fibronectin, and keratin (Boster, Pleasanton, CA, USA). After washing, cells were incubated with secondary antibodies (Abbkine, Wuhan, China), followed by sealing with anti-fade fluorescence mounting medium containing DAPI (Servicebio, Wuhan, China). Immediately, samples were pictured with fluorescent microscope (OLYMPUS, Tokyo, Japan).

### 4.3. CCK-8 Assay 

Rb3 powder was purchased from Aladdin Chemicals (purity ≥ 98%, Aladdin Chemicals, Shanghai, China). There were 10 groups with 6 wells per group set up by treating with different concentrations of Rb3 (0, 50, 100, 200 and 400 μM) with or without 1 μg/mL *P. gingivalis* LPS (InvivoGen, Shanghai, China) stimulation. HPLCs were cultivated for 24 h and incubated with 10% CCK-8 solution (Dojindo, Kumamoto, Japan) for 60 min, followed by optical density (OD) determination. 

### 4.4. ELISA

HPLCs were pretreated 1 h with Rb3 (0, 25, 50 and 100 μM) and stimulated with 1 μg/mL *P. gingivalis* LPS for 24 h or 4 d. Culture media were collected and detected with ELISA kit (NeoBioscience, Shenzhen, China) following manufacturer’s instructions. Cells were collected with TRIZON reagent (CWBio, Beijing, China) at the same time and stored at −80 °C for the subsequent mRNA examination.

### 4.5. RNA Extraction, Reverse Transcription and qPCR Analysis

Total RNA were extracted with an RNA extraction Kit (CWBio, Beijing, China) and quantified. Equivalent amounts of RNA (1 μg) were used for reverse transcription (Vazyme, Nanjing, China). qPCR was performed using SYBR qPCR Mix (Vazyme) and gene specific primers (IL-6, IL-1β, IL-8, TLR2, TLR4 and GAPDH, Table 1) according to manufacturer’s instruction. Expression of target genes were normalized to GAPDH.

### 4.6. Network Pharmacological Analysis of Rb3

To predict the potential target of Rb3 in human beings, network pharmacological analysis was applied. The overall analysis workflow is summarized in Figure 2A, which initiates from PubChem.

### 4.7. Western Blot

HPLCs were pretreated with Rb3 (0, 50,100 μM) for 60 min, followed by stimulating with *P. gingivalis* LPS (1 μg/mL) for 30 min. Cells were lysed with RIPA buffer (Beyotime, Shanghai, China), sonicated and centrifuged. Samples were quantified with Bicinchoninic acid kit (Beyotime) and normalized. A 20 μg of proteins was separated with electrophoresis and electro-transferred to polyvinylidene difluoride membrane (Millipore, Billerica, MA, USA). Membranes were blocked, and then incubated with primary antibodies recognizing p38, JNK, ERK, AKT, p65 NF-κB, p-p38, p-JNK, p-ERK, p-AKT, p-p65 NF-κB and GAPDH proteins (all from CST, Danvers, MA, USA), followed by incubating with secondary antibody (CST), and visualization using AdvanBlock™-Chemi reagent. The chemiluminescent signal was captured by Image Studio System (LICOR, Lincoln, NE, USA).

### 4.8. Animal and Experimental Design 

This experiment was approved by the Institutional Animal Care and Use Committee of Wuhan University (S07919070B). Sixteen 10-week-old male SD rats with weight 280 to 300 g were randomly assigned into four experimental groups (4 rats/group): control group, sham group, LPS group, and LPS + Rb3 group. Experimental periodontitis rats were built up by gingival injection of *P. gingivalis* LPS (1 mg/mL) around the neck of first upper molar under isoflurane respiratory anesthesia. Sham group received injections of vehicle (normal saline) in the same way. Control group received no treatment. G-Rb3 powder was dissolved in DMSO and diluted to 100 μM with normal saline, containing approximately 0.1% DMSO. Except for controls, animals received gingival injection of G-Rb3 (LPS + Rb3 group) and 0.1% DMSO in normal saline (LPS and sham group) at the same site as LPS/normal saline injections. This experiment lasted for 20 days, and injection of LPS was operated on odd days like 1, 3, 5, and injection of Rb3 was operated on even days like 2, 4, 6, based on several previous methods with modification [42,43,44,45]. 

### 4.9. Macroscopic Measurement of Alveolar Bone Loss

Rats were sacrificed and maxilla, together with three molars on each side, were fixed. The right half were stained with methylene blue and pictured under stereomicroscope [46]. Seven palatal distances (illustrated in Figure 6A) between CEJ and AC were measured, and sum of two/three distances from each molar was adopted as a measurement of ABL [47]. 

### 4.10. Assessment of Bone Resorption Using Micro-CT

Micro-CT images of the right halves were taken with Skyscan (Bruker MicroCT, Kontich, Belgium) after macroscopic examination. Three-dimensional reconstructions were made and analyzed by NRecon 1.6.3 (Brucker MicroCT) [48]. 

### 4.11. H&E and TRAP Staining

The left jaw of rats were decalcified and sectioned (5 μm) in the mesio-distal direction as previously [49]. Then specimens were stained with H&E and TRAP (Wako Pure Chemical Industries, Osaka, Japan) as manufacturer’s instructions. All staining slides in this experiment were scanned and analyzed using Slide Converter 2.3 (3D HISTECH, Budapest, Hungary). For comparison of osteoclasts genesis, an area of interest (AOI) with standardized dimensions was prepared and the upper line of AOI was placed at the level of AC of the mesial molar in each slide, to allow equal comparisons of the quantified alveolar bone; active osteoclasts were defined as multinucleated (≥2) TRAP-positive cells in contact with the bone surface; the number of osteoclasts per square millimeter of alveolar bone was calculated and used for statistical analysis [50].

### 4.12. IHC Assay 

IHC was performed to determine the expression of TLR2 in rat periodontal tissues according to manufacturer’s protocol (Zhong Shan Biotech, Beijing, China). Briefly, specimens were processed with gastric enzyme, blocked, and then incubated with primary antibody against TLR2 (CST) and biotinylated secondary antibody, followed by staining using a DAB kit (Maixin Biotechnology, Fuzhou, China). 

### 4.13. Statistical Analysis for Cell and Animal Studies

All values were presented as means ± standard error (SE). Unpaired two tailed Student’s *t*-test was adopted to compare differences between two groups and *p* < 0.05 were considered significant. Statistical analyses were performed using GraphPad Prism 5.0 software. 

## 5. Conclusions

The anti-inflammatory effect of Rb3 was reported for the first time in HPLCs and rat models. The results from the present work clearly suggested that Rb3 might exert a protective effect against *P. gingivalis* LPS-induced inflammation closely related to the inhibition of MAPK/AKT/NF-κB signaling pathways and attenuated alveolar bone resorption in experimental periodontitis rats. This observation is consistent with the concept of host modulatory therapy, implying that Rb3 may potentially be used for modulating the host response in the treatment of inflammatory diseases, such as periodontitis caused by *P. gingivalis*. Further clinical study is necessary to investigate the effectiveness of Rb3 as an adjuvant therapy in the treatment of chronic periodontitis. 

## Figures and Tables

**Table 1:** Oligonucleotide Primers Used for qPCR.

Primer	Sequence	Amplicon Size (bp)	NCBI Gene ID
GAPDH	Forward 5′-CAGGAGGCATTGCTGATGAT-3′	138	2597
	Reverse 5′-GAAGGCTGGGGCTCATTT-3′		
IL-6	Forward 5′-CACTGGTCTTTTGGAGTTTGAG-3′	101	3569
	Reverse 5′-GGACTTTTGTACTCATCTGCAC-3′		
IL-1β	Forward 5′-GCCAGTGAAATGATGGCTTATT-3′	85	3553
	Reverse 5′-AGGAGCACTTCATCTGTTTAGG-3′		
IL-8	Forward 5′-AACTGAGAGTGATTGAGAGTGG-3′	147	3576
	Reverse 5′-ATGAATTCTCAGCCCTCTTCAA-3′		
TLR2	Forward 5′-AAGCAGCATATTTTACTGCTGG-3′	184	7097
	Reverse 5′-CCTGAAACAAACTTTCATCGGT-3′		
TLR4	Forward 5′-GACTGGGTAAGGAATGAGCTAG-3′	143	7099
	Reverse 5′-ACCTTTCGGCTTTTATGGAAAC-3′		

## Supplementary Materials

The following are available online, Figure S1: Potential target candidates of Rb3 in mitogen-activated protein kinase (MAPK) signaling pathway from GO analysis, which are highlighted (in red box).
