# Supplementary material for: Ginsenoside Rb3 Inhibits Pro-Inflammatory Cytokines via MAPK/AKT/NF-κB Pathways and Attenuates Rat Alveolar Bone Resorption in Response to Porphyromonas gingivalis LPS
Source: Molecules. 2020 Oct 20;25(20):4815. doi: 10.3390/molecules25204815 (PMC7588009; doi:10.3390/molecules25204815)
Supplement: Supplementary file 1 [file molecules-25-04815-s001.pdf]

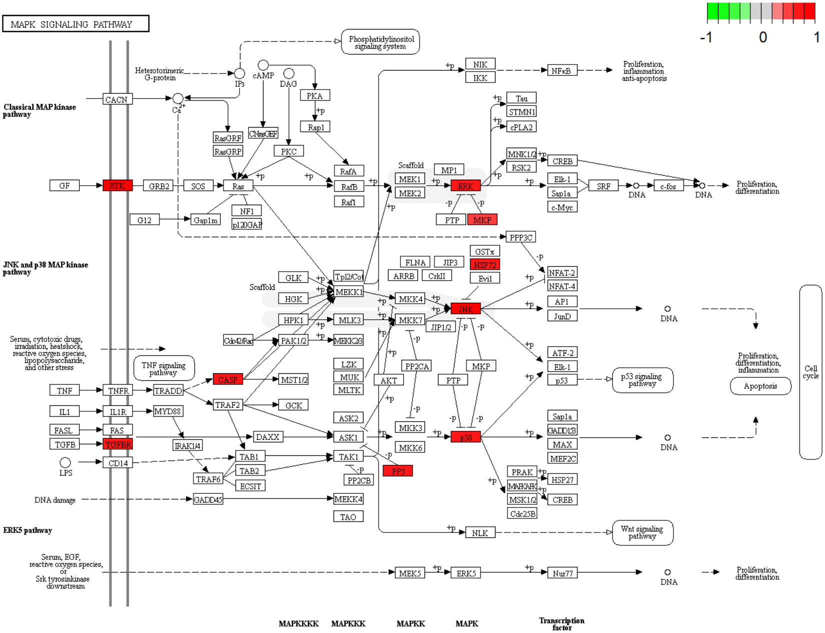

Data on KEGG graph
Rendered by Pathview
